# Investigating the Determinants of High Case-Fatality Rate for Coronavirus Disease 2019 in Italy

**DOI:** 10.1017/dmp.2020.106

**Published:** 2020-04-16

**Authors:** Francesco Barone-Adesi, Luca Ragazzoni, Maurizio Schmid

**Affiliations:** Department of Translational Medicine, University of Eastern Piedmont, Novara, Italy; CRIMEDIM – Research Center in Emergency and Disaster Medicine, University of Eastern Piedmont, Novara, Italy; Department of Engineering, Roma Tre University, Rome, Italy

**Keywords:** epidemiologic methods, infection control, public health

## Abstract

Case-Fatality Rate (CFR) for COVID-19 in Italy is apparently much higher than in other countries. Using data from Italy and other countries we evaluated the role of different determinants of this phenomenon. We found that the Italian testing strategy could explain an important part of the observed difference in CFR. In particular, the majority of patients that are currently tested in Italy have severe clinical symptoms that usually require hospitalization and this translates to a large CFR. We are confident that, once modifications in the testing strategy leading to higher population coverage are consistently adopted in Italy, CFR will realign with the values reported worldwide.

The epidemiologic features of the recent pandemic of coronavirus disease 2019 (COVID-19) have been shown to relevantly vary among countries,^1^ with substantial differences in terms of incidence, mortality, and case-fatality rate (CFR), a parameter whose crude estimation is obtained by dividing the number of deaths by the total number of cases. In particular, a substantially higher CFR for COVID-19 was reported for Italy compared with other countries, and several authors put forward different hypotheses to explain it.^2-4^ Using data from China and Italy, 1 paper suggested that this phenomenon could be due to mainly 3 different factors^2^: (1) a diverse age distribution; (2) a different definition of COVID-19-related deaths; and (3) a country-specific strategy in testing. Expanding on this, we tried to single out the effect coming from each of the 3 suggested causes. We used direct standardization and applied Chinese age-specific risk displayed in the Onder et al. study^2^ to the Italian age structure to accommodate the possible effect of age. As expected, the corresponding standardized CFR was higher than that shown in the original article (5.3% as compared with 2.4%). However, it was still far from the 1 currently reported in Italy (12.7%), thus suggesting that the different age distribution between the 2 samples accounts for only a part of the difference in the CFR. Moreover, this would hardly explain why the Italian CFR is also substantially higher than that of countries, such as Greece, Portugal, and Germany, which share a very similar age structure with the Italian population. Regarding the definition of COVID-19-related deaths, the criteria used by each country are often not clearly defined, making comparisons difficult. However, this might not apply to the comparison between Italian and Chinese data used in the Onder et al. study,^2^ because all confirmed cases of patients who eventually died were considered as COVID-19-related deaths in China, a definition very similar to that used in Italy.

On the other hand, we think that the Italian testing strategy could explain an important part of the observed difference in CFRs. The majority of patients who are currently tested in Italy have severe clinical symptoms that usually require hospitalization. Indeed, the proportion of positive patient-cases that are admitted to the hospital in Italy is about 40% (and used to be much higher in the past weeks),^5^ whereas it was about 10–20% in China.^6^ As the positive cases resulting from this testing strategy are so skewed toward more serious conditions, it is not surprising that such a high CFR is observed. This phenomenon could also explain the substantial heterogeneity of CFRs observed among Italian regions, which can independently determine their own testing strategy. Figure 1 shows a clear association between the proportion of hospitalized cases and CFRs. We are confident that, after modifications in the testing strategy leading to higher population coverage will be consistently adopted in Italy, CFRs will realign with the values reported worldwide.

**FIGURE 1:**
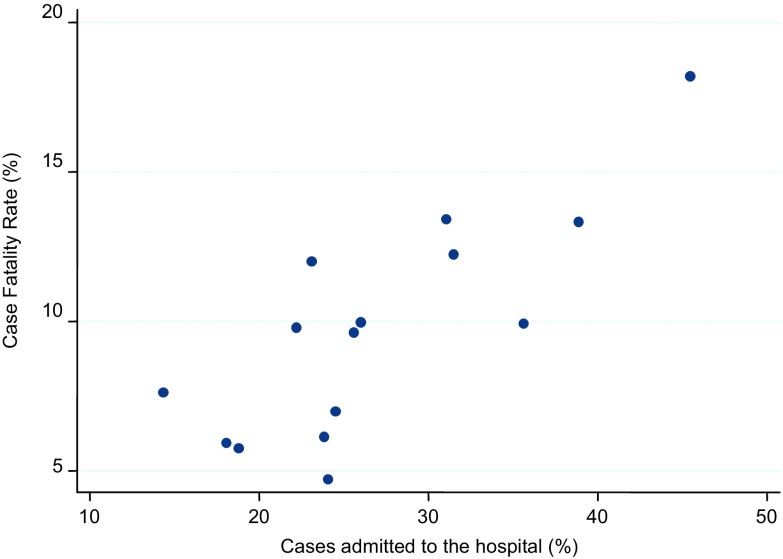
Relation Between the Proportion of Cases Admitted to the Hospital and Case-Fatality Rate in Different Italian Regions. (Only Regions With Mortality Rates for COVID-19 Above 1 per 10 000 Inhabitants are Reported.)
